# Hepatocarcinogenesis in transgenic mice carrying hepatitis B virus pre-S/S gene with the sW172* mutation

**DOI:** 10.1038/oncsis.2016.77

**Published:** 2016-12-05

**Authors:** M-W Lai, K-H Liang, W-R Lin, Y-H Huang, S-F Huang, T-C Chen, C-T Yeh

**Affiliations:** 1 Division of Pediatric Gastroenterology, Department of Pediatrics, Chang Gung Memorial Hospital, Linkou, Taiwan; 2 Liver Research Center, Department of Hepato-Gastroenterology, Chang Gung Memorial Hospital, Linkou, Taiwan; 3 Molecular Medicine Research Center, Chang Gung University, Taoyuan, Taiwan; 4 Institute of Molecular and Genomic Medicine, National Health Research Institutes, Zhunan, Taiwan; 5 Department of Pathology, Chang Gung Memorial Hospital, Linkou, Taiwan

## Abstract

Hepatitis B virus (HBV) carrying the rtA181T/sW172* mutation conferred cross-resistance to adefovir and lamivudine. Cell-based and clinical studies indicated that HBV carrying this mutation had an increased oncogenic potential. Herein, we created transgenic mouse models to study the oncogenicity of the HBV pre-S/S gene containing this mutation. Transgenic mice were generated by transfer of the HBV pre-S/S gene together with its own promoter into C57B6 mice. Four lines of mice were created. Two of them carried wild-type gene and produced high and low levels of HBV surface antigen (HBsAg) (TgWT-H and L). The other two carried the sW172* mutation with high and low intrahepatic expression levels (TgSW172*-H and L). When sacrificed 18 months after birth, none of the TgWT mice developed hepatocellular carcinoma (HCC), whereas 6/26 (23.1%) TgSW172*-H and 2/24 (8.3%) TgSW172*-L mice developed HCC (TgWT vs TgSW172* *P*=0.0021). Molecular analysis of liver tissues revealed significantly increased expression of glucose-regulated protein 78 and phosphorylated extracellular signal-regulated kinases 1 in TgSW172* mice, and decreased expression of B-cell lymphoma-extra large in TgSW172*-H mice. Higher proportion of apoptotic cells was found in TgSW172*-H mice, accompanied by increased cyclin E levels, suggesting increased hepatocyte turnover. Combined analysis of complimentary DNA microarray and microRNA array identified microRNA-873-mediated reduced expression of the CUB and Sushi multiple domains 3 (CSMD3) protein, a putative tumor suppressor, in TgSW172* mice. Our transgenic mice experiments confirmed that HBV pre-S/S gene carrying the sW172* mutation had an increased oncogenic potential. Increased endoplasmic reticulum stress response, more rapid hepatocyte turnover and decreased CSMD3 expression contributed to the hepatocarcinogenesis.

## Introduction

Worldwide, hepatocellular carcinoma (HCC) is the sixth most commonly diagnosed solid cancer and the second most common cause of cancer-related death.^1^ In Taiwan, it ranked the first and second respectively in male and female cancer mortality.^2^ HCC is multifactorial in origin and the three most important causes of liver cancer are chronic hepatitis B virus (HBV) infection, chronic hepatitis C virus infection and alcoholic liver disease.^3, 4^ Other risk factors include old age, male gender, underlying chronic liver diseases, liver cirrhosis, aflatoxin exposure and diabetes.^5, 6^ In Southeast Asia, chronic hepatitis B is highly prevalent, leading to a correspondingly high incidence of HCC. Despite a successful vaccination program, HBV remains the major etiological factor of HCC in Taiwan to date.

Because of a multifactorial etiology, the key molecular pathways leading to hepatocarcinogenesis are not well understood. It is believed that hepatocarcinogenesis involved not only multiple steps of molecular events but also heterogeneous cellular pathways.^7, 8, 9^ In HBV-associated HCC, several virological factors have been demonstrated to manifest important prognostic correlation. For example, high serum or intrahepatic HBV-DNA levels and the presence of basal core promoter A1762T/G1764A mutation can independently predict outcomes of long-term chronic HBV infection as well as postoperative survival in HCC.^10, 11, 12^ In addition, antiviral therapy using nucleos(t)ide analogs to suppress HBV-DNA levels significantly reduced long-term HCC occurrence and postoperative HCC relapse.^13, 14, 15^

Despite the success of antiviral strategy, several studies indicated that in cirrhosis patients, anti-HBV therapy with nucleos(t)ide analogs cannot completely eliminate HCC occurrence.^16, 17^ One of the explanations for such observation is the failure to completely suppress HBV-DNA replication. Our groups have shown that potent anti-HBV treatment frequently leads to selection of the S gene mutants.^18, 19^ Such mutants could be generated due to emergence of the overlapping polymerase resistant mutations or alternatively, by an enhanced humoral immunity. Of them, the S gene truncation mutants, such as the rtA181T/sW172* mutant, were associated with hepatocarcinogenesis.^20, 21^ It has been suggested that intra-hepatic accumulation of mutant pre-S/S proteins due to secretion defect could be the reason why such mutants were selected during antiviral therapy and why they were associated with HCC.^20, 21, 22, 23, 24^ Other supporting evidence included previous studies showing that S gene stop codon mutations found in the integrated HBV genomes in HCC tissues harbored transactivation and oncogenic activities.^25, 26^ However, hepatic expression levels of the S truncation proteins derived from HBV integration was usually low and the sW172* mutation was not located on the characterized ‘transactivation-on' region. On the other hand, HBV carrying the rtA181T/sW172* mutation could accumulate to a high serum level, insuring the generation of significant amount of truncated pre-S/S proteins.^27^ Clinical analysis showed that the rtA181T/sW172* mutation was associated with an increased risk of HCC in cirrhotic patients.^28^ As such, this truncated pre-S/S protein is considered an oncogenic hepatitis B viral protein in review articles.^29, 30^

In the present study, we aimed to provide transgenic mouse evidence for the increased oncogenicity of pre-S/S proteins carrying the rtA181T/sW172* mutation. Such experiment could illustrate that the truncated pre-S/S proteins alone contribute to an increase of oncogenicity, excluding the interference effect of HBV-DNA level and other HBV viral proteins.

## Results

### Establishment of transgenic mice carrying HBV pre-S/S gene

**Figure 1:**
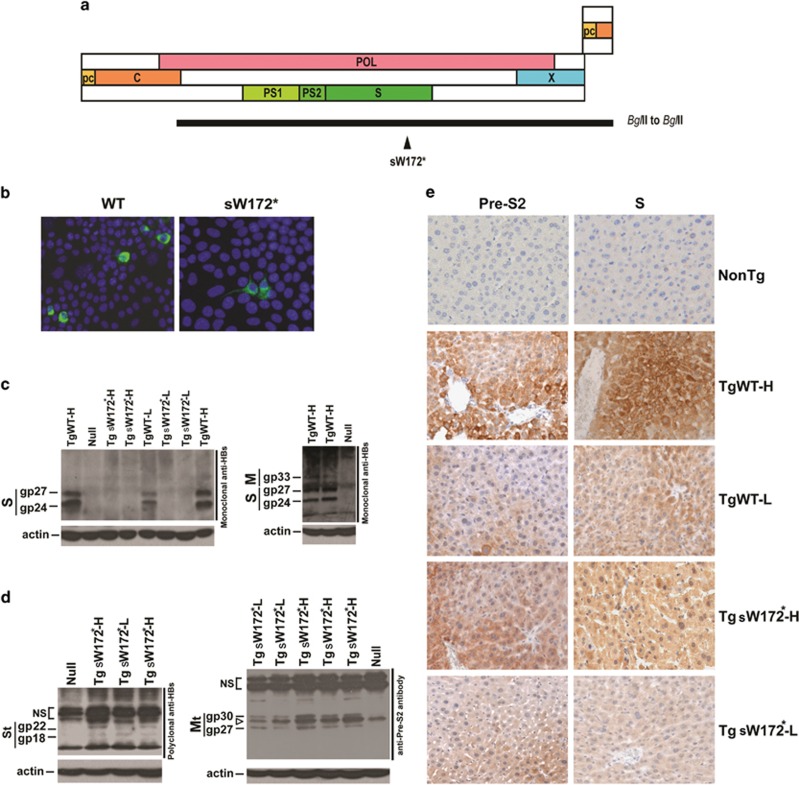
Genomic structure of the transgenes and expression of the pre-S/S wild type and mutant proteins in the transgenic mice. (**a**) Genomic structure of the HBV pre-S/S transgenes. PC, precore region (yellow); (**c**) core region (orange); POL, polymerase (pink); X, X region (blue); PS1/PS2/S, pre-S1/pre-S2/surface region (light, medium, dark green); Horizontal solid bar, *Bgl*II to *Bgl*II HBV DNA fragments as the transgenes. Triangle, the point mutation leading to sW172* mutation. (**b**) Transient transfection using the plasmids carrying the transgenes to transfect Hepa1-6 mouse hepatoma cells before generation of transgenic mice. (**c**) Western blot analysis to detect wild type pre-S/S protein expression using monoclonal anti-HBs antibodies. Right panel, over-exposure to visualize middle surface protein. S, small surface proteins (gp24 and gp27); M, middle surface proteins (only gp33 was clearly visible, gp36 was barely seen (not marked)). (**d**) Western blot analysis to detect mutant pre-S/S protein using polyclonal anti-HBs antibodies (left panel) or anti-pre-S2 antibodies (right panel). St, truncated surface proteins (sW172* truncation, gp18 and gp22); NS, non-specific bands; Mt, truncated middle surface proteins (gp27 and gp30); Empty arrowhead, a non-specific band also found in null mice. Molecular weights of all protein are obtained through electrophoresis of a molecular weight marker in parallel. (**e**) IHC analysis for pre-S/S protein expression. Left, anti-pre-S2 antibody is used for detection; right, polyclonal anti-S antibody is used. NonTg, non-transgenic control mice.

Before generation of transgenic mice, the plasmids carrying the wild type and mutant pre-S/S gene were transiently transfected into Hepa1-6 cells to insure the endogenous HBV pre-S/S promoter sufficiently driven the expression of pre-S/S proteins (Figure 1b). The method to generate transgenic mice carrying HBV pre-S/S gene was described in the Materials and methods section. Four lines of mice were generated (Table 1). TgWT-L and TgWT-H carried wild-type pre-S/S gene and expressed low and high levels (419±162 IU/ml versus 599± 159 IU/ml) of serum HBsAg, respectively. TgSW172*-L and TgSW172*-H carried HBV pre-S/S gene containing the sW172* mutation and expressed low and high levels of intrahepatic HBsAg respectively, determined by western blot and immunohistochemistry (IHC) analysis (Figures 1c–e). Low level of serum HBsAg (10.2±19.1 IU/ml) was detected in TgSW172*-H mice, whereas serum HBsAg was undetectable in TgSW172*-L mice. Western blot analysis using monoclonal antibody against HBsAg detected small surface (S) proteins, gp24 and gp27, only in TgWT-L and H mice but not in TgSW172*-L and H mice (Figure 1c, left panel). When the film was overexposed, the middle surface protein (M), gp33, was visualized (Figure 1c, right panel). The gp36 was only barely seen (not marked). However, large surface proteins could not be clearly demonstrated in this study. To detect truncated surface proteins, polyclonal antibody against HBsAg was used. Small amounts of truncated S proteins (St), gp18 and gp22, were detected (Figure 1d, left panel) in TgSW172*-L and H mice. When pre-S2 antibody was used, truncated M proteins, gp27 and gp30, were visualized (Figure 1d, right panel). A larger protein corresponding to the truncated large surface protein was seen in some but not all mice (Figure 1d, right panel, under the non-specific bands). The polyclonal anti-Pre-S2 was raised against the pre-S2 region, which was absent in the major S protein. Detection using the polyclonal anti-HBs, unfortunately, displayed heavy nonspecific (NS) bands over the predicted wild type HBsAg positions (Figure 1d). IHC was performed to cross-examine the surface protein expression (Figure 1e). The results were consistent with the western blot data.

Copy number of the transgene was determined by real-time PCR method. It was found that each of the four lines carried only one copy of transgene.

### Development of HCC in TgSW172* mice

All mice were sacrificed at 18 months of age. Liver was examined for grossly visible tumors. Parts of the tumors were embedded in formalin and processed with haematoxylin and eosin stain (H & E) for pathological examination. IHC staining for glypican-3 was performed for additional verification. In total, 27, 27, 24 and 26 transgenic mice (TgWT-L, TgWT-H, TgSW172*-L and TgSW172*-H, respectively) were examined. It was found that no HCC developed in TgWT-H and L mice, whereas 2 (8.3%) and 6 (23.1%) of TgSW172*-L and TgSW172*-H mice, respectively, developed HCC (TgWT versus TgSW172*, *P*=0.0021) (Table 1).

### Increased ER stress response and hepatocyte turnover in HBV pre-S/S transgenic mice

**Figure 2:**
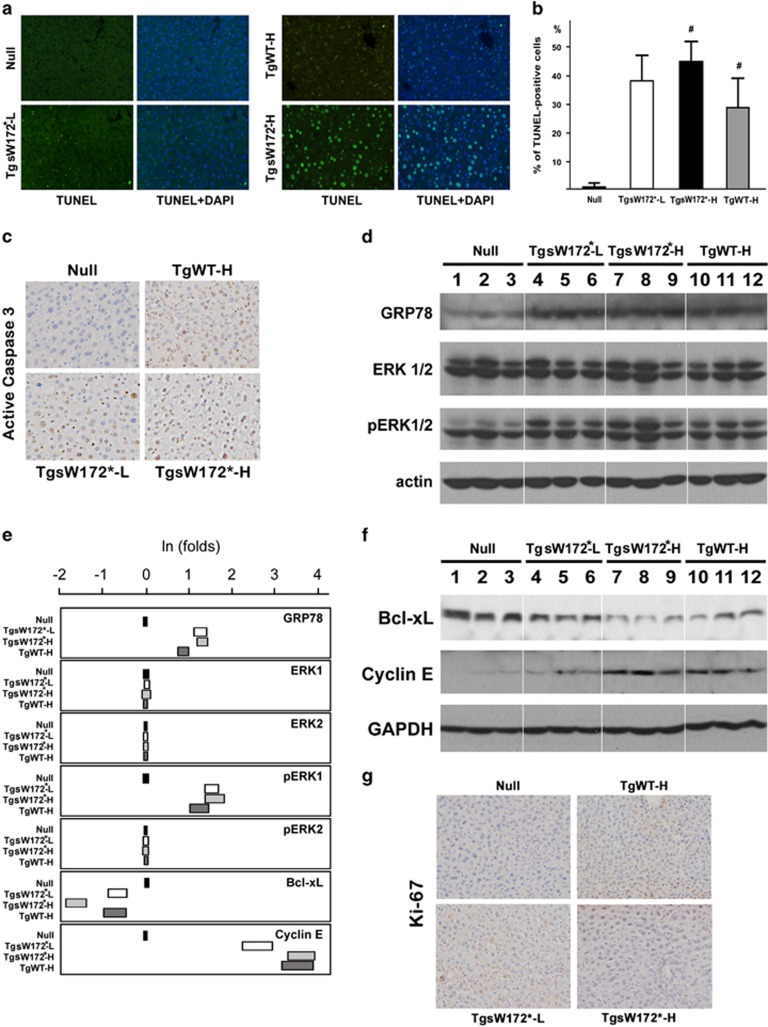
Apoptosis and signaling protein expression in transgenic mice. (**a**) TUNEL assay to evaluate the percentages of apoptotic cells in transgenic mice. (**b**) Comparison of the percentages of positively stained apoptotic cells in TUNEL assay. Empty bar, TgSW172*-L mice; solid bar, TgSW172*-H mice; gray bar, TgWT-H mice. ^#^*P*<0.05. (**c**) IHC staining of active caspase 3 in transgenic mice. (**d–f**) Comparison of signaling protein expression levels in transgenic mice. Results of western blot analyzes were shown (**d**, **f**). The first measured expression level for each protein in the null mice was assigned as 1-fold. All other levels were calculated as folds of difference with reference to this level. The natural logarithms (ln) of the fold numbers were depicted for comparison (**e**). (**g**) IHC staining of Ki-67 in transgenic mice.

To explore mechanisms of hepatocarcinogenesis in the transgenic mice, several assays were carried out. TUNEL assay to estimate the numbers of apoptotic cells in liver showed a significantly higher percentage of apoptotic cells in TgsW172*-H mice compared with that in TgWT-H mice (Figures 2a and b). IHC analysis for active caspase 3 expression was also performed for confirmation (Figure 2c). Consistently, a higher percentage of positive cells was found for all three Tg mice compared with that of the null mice and a higher percentage was observed for TgsW172*-H mice compared with that of TgWT-H mice. Semi-quantitative assessment using western blot analysis showed an increase of GRP78 expression suggesting an increase of endoplasmic reticulum (ER) stress (Figures 2d and e). Decrease of B-cell lymphoma-extra large expression was also found in these transgenic mice (Figures 2e and f). On the other hand, an increase of cyclin E and phosphorylated extracellular signal-regulated kinases 1 expression levels was found, suggesting activation of proliferation signal and increased cell proliferation (Figures 2e and f). IHC analysis for Ki-67 expression also confirmed the results (Figure 2g).

### Search for microRNA-mediated differentially expressed genes between transgenic mice carrying wild type and mutant pre-S/S gene.

**Figure 3:**
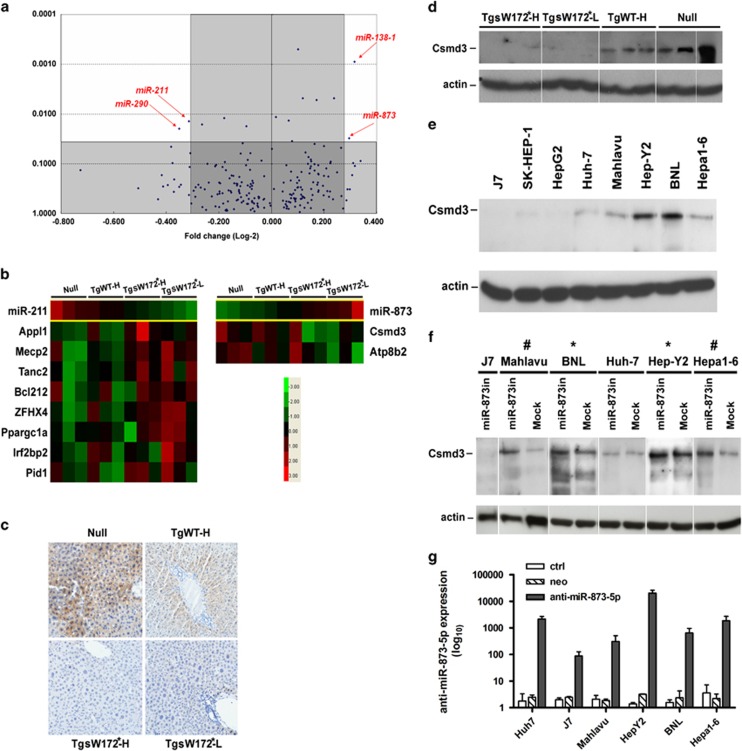
Co-analysis of cDNA microarray and microRNA array in transgenic mice. (**a**) The distribution of the fold changes (horizontal axis) and *P-*values (vertical axis) of microRNAs differentially expressed between the TgWT-H and TgSW172*-L mice (three mice from each line were assayed). Four microRNAs (miR-211, miR-290, miR-138-1 and miR-873) are selected for subsequent cDNA microarray matching. Only miR-211 and miR-873 have matched up- and down-regulated targets in the cDNA microarry assays. (**b**) Gene expression level changes of miR-211 and miR-873 targeted genes in null, TgWT-H, TgSW172*-H and TgSW172*-L mice. (**c**) IHC analysis of CSMD3 protein expression in hepatocytes. (**d**) Western blot analysis and of the CSMD3 protein expression in null, TgWT-H, TgSW172*-H and TgSW172*-L mice. (**e**) Expression levels of CSMD3 in liver cancer cell lines. (**f**) CSMD3 protein level changes on miR-873 knock-down in liver cancer cell lines. ^#^*P*<0.005. *P<0.05. (**g**) GRP78 expression in miR-873 over-expressed liver cancer cell lines.

Despite a significantly increased number of apoptotic cells in TgSW172*-H mice compared with that of TgWT-H mice (Figure 2a), both lines shared similar profiles of ER stress response, increased apoptosis and increased hepatocyte proliferation. It was difficult to explain the 23.1% versus 0% of difference in HCC development. To search for other differential molecular pathways related to hepatocarcinogenesis, we performed two microarray assays: microRNA array and complimentary DNA (cDNA) microarray. This experimental strategy was aimed to identify a microRNA-mediated regulatory pathway shared by both human and mice, and to minimize the number of false positive targets. After eliminating the microRNAs that did not have human equivalents, the distribution of fold-change and statistical significance of the remaining candidates were depicted in Figure 3a. Only four microRNAs (microRNA-211, 290, 138-1 and 873) had the highest fold-change and the difference was statistically significant (*P*<0.05). Subsequently, the data derived from these two arrays were matched to identify two sets of targets: down-regulated microRNAs with matched up-regulated target genes (Figure 3b, left panel) and up-regulated microRNA with matched down-regulated target genes (Figure 3b, right panel). Only microRNA-211 and micriRNA-873 (miR-873) satisfied these criteria. Real-time PCR (RT-qPCR) was carried out to verify the up- and downregulation of the identified supposedly dysregulated genes using an independent cohort of transgenic mice. The results showed that only upregulation of miR-873 and downregulation of its putative target genes, *CSMD3* (CUB and Sushi multiple domains 3) and *ATP8B2* (ATPase, aminophospholipid transporter, class I, type 8b, member 2), were consistently verified. Because *CSMD3* had been implicated as a cancer-related gene in previous reports, we selected this gene for further study. On the other hand, we could not obtain suitable antibodies against ATP8B2 for clear western blot and IHC analysis (data not shown). Western blot analysis confirmed CSMD3 downregulation in TgSW172*-H and L mice, but not in TgWT-H mice (Figure 3c). IHC analysis showed consistent results (Figure 3d). Finally, inhibition of miR-873 expression was achieved using a lentivirus-based vector in various hepatoma cell lines. Before inhibition experiments, the baseline CSMD3 expression was undetectable or barely detected in J7, SK-Hep-1 and HepG2 cells. Following miR-873 inhibition, the *CSMD3* expression remained undetectable in these three cell lines. However, marked upregulation (5–10 folds) of CSMD3 was found in Mahlavu and Hepa1-6 cells and mild increase (1.5 to 2 folds) was found in BNL and Hep-Y2 cells. No significant change was found in Huh7 cells (Figures 3e and f). The efficiency of anti-miR-873 expression was also assessed for comparison (Figure 3g).

**Figure 4:**
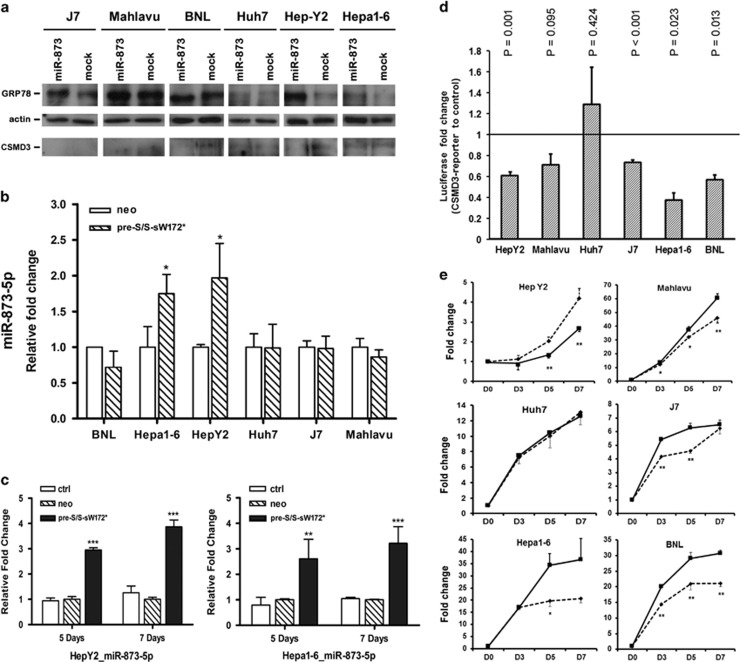
Regulatory cascade from pre-S/S-sW172*, miR-873, to *CSMD3*. (**a**) Western blot analysis for GRP78 and CSMD3 expression in hepatoma cells with or without miR-873 overexpression. (**b**) Enhanced miR-873-5p expression following expression of the pre-S/S-sW172* protein in hepatoma cell lines. Empty bars, mock transfection; shaded bars, hepatoma cells expressing the pre-S/S-sW172* protein. (**c**) Validation of increased miR-873-5p expression in Hep-Y2 and Hepa1-6 cells after pre-S/S-sW172* expression. Empty bars, no transfection; shaded bars, mock transfection; solid bars, cells expressing pre-S/S-sW172* protein. (**d**) Luciferase reporter assay using the 3'-UTR of *CSMD3* gene. The fold-change of expression levels following miR-873 expression in hepatoma cell lines were represented by gray bars. Statistical significance was calculated and the *P-*value was given on top of each bar. (**e**) MTT assays for cell proliferation rates in hepatoma cells expressing miR-873 (solid lines) and mock controls (dashed lines). *P<0.05; **P<0.005.

On the other hand, when miR-873 was overexpressed, suppression of *CSMD3* expression was observed in Mahlavu, BNL and Hep-Y2 cells. Mild suppression (0.8-fold only) was observed for Hepa1-6 cells (Figure 4a). CSMD3 was undetectable in J7 cells with or without miR-873 overexpression. To understand whether the increased ER stress in transgenic mice was caused by pre-S/S-sW172* alone or also by miR-873 upregulation, the GRP78 levels were also compared between hepatoma cells with or without miR-873 overexpression. The results showed significant increase of GRP78 expression in J7, Mahlavu, Hep-Y2 and Hepa1-6 cells (Figure 4a).

### Activation of miR-873 to target *CSMD3* by pre-S/S-sW172* expression in hepatoma cell lines

To further clarify the regulation cascade, pTg-sW172* was transfected into BNL, Hepa1-6, HepY2, Huh7, J7, and Mahlavu cells and miR-873 was assayed 3 days after transfection. Up-regulation of miR-873 was found in Hepa1-6 and HepY2 cells (Figure 4b). Independent transfection experiments were preformed for these two cell lines and the cells were harvested 5 and 7 days after transfection. Increasing levels of miR-873 upregulation were noted (Figure 4c).

To understand whether *CSMD3* was an authentic target of miR-873, the 3′-UTR of *CSMD3* gene was inserted into the 3′-UTR of a luciferase gene for miR-873 co-transfection experiments. It was found that in HepY2, J7, Hepa1-6 and BLN cells, *CSMD3* 3′-UTR-associated luciferase activities were significantly down-regulated (Figure 4d). All downregulations were disrupted when the presumed recognition seed sites were mutated.

These results indicated that pre-S/S-sW172* could upregulate miR-873 expression in at least two hepatoma cell lines (Hepa1-6 and HepY2). Subsequently, miR-873 could target at *CSMD3* by binding to the 3′-UTR of its transcripts. This occurred in all hepatoma cell lines tested, except for Huh7 cells. These results were consistent with the actual changes of CSMD3 protein levels upon miR-873 inhibition (Figure 3f) and overexpression (Figure 4a).

Finally, the effect of miR-873 on cell growth was examined by MTT (3-(4,5-dimethylthiazol-2-yl)-2,5-diphenyltetrazolium bromide) assay. It was found that overexpression of miR-873 led to enhanced cell proliferation in Mahlavu, J7, Hepa1-6 and BNL cells. However, it had no effect on Huh7 cells and it had a growth-inhibitory effect on HepY2 cells (Figure 4e).

## Discussion

The rtA181T/sW172* mutant exerts cross-resistance to both lamivudine and adeforvir.^31, 32^ In lamivudine-resistant patients receiving adefovir rescue therapy, about 8–20% of patients will eventually develop cross-resistance.^32^ In Taiwan and Hong Kong, this problem is gradually overcome since lamivudine is rarely used as the first-line antiviral drug at this time. Instead, nucleot(s)ide analogs with high resistance barrier, such as entecavir, are being widely used. The situation, however, remains critical in China, where low-cost, generic adefovir is generally used as the first-line rescue drug for lamivudine, resulting in emergence of cross-resistance in a significant proportion of patients. The actual prevalence of rtA181T/sW172* mutant in China is unknown,^33^ nor do we know whether it is capable of transmission among people.

S gene truncation can develop not only in lamivudine and adefovir-treated patients but also in peginterferon-treated patients, vaccine failure patients and antiviral treatment naïve patients.^19, 20^ Recent studies using HBcAg/HBsAg-postive HCC tissues uncovered another frequent S gene truncation mutation, sW182*.^34^ It has been reported to be highly prevalent in Korean HCC patients and it has been found in HBV and HIV coinfected patients.^35, 36^ Tumorigenicity assessment in nude mice showed an even higher oncongenicity.^21^ Taken together, our transgenic mice model can serve as a representative tool to study molecular pathways of hepatocarcinogenesis originated from the S gene truncation mutation.

From the results in our search for oncogenic mechanisms, we speculated increase of ER stress response accompanied by increased apoptotic hepatocytes was followed by increased compensatory liver cell proliferation and regeneration. This scenario has been observed in other HBV-related transgenic models developing HCC.^37, 38, 39^ In the present study, however, we found that these events also occurred in TgWT-H mice, in which no HCC developed. Such discrepancy urged us to search for other differentially regulated pathways between TgWT and TgSW172*. To avoid misidentification of insignificant background genes and to focus on microRNA-mediated dysregulation, we used two-array assays for cross reference. After verification process, only miR-873-mediated downregulation of CSMD3 expression was consistently confirmed in an independent cohort of TgSW172* mice. Inhibition of miR-873 expression resulted in upregulation of CSMD3 in several hepatoma cell lines, indicating that CSMD3 is indeed a target of miR-873. According to the Cancer Atlas of the Human Protein, CSMD3 are expressed in low to medium levels in the cytoplasm/membrane of 20 different cancer tissues. In 12 HCC tissues, CSMD3 is undetectable in five and weakly positive in four.^40, 41^ CSMD3 has been found to have a high mutation rate and a low expression level in lung cancer, ovarian cancer and several other cancers and thus possibly has a tumor suppressor function.^42, 43, 44, 45, 46^ CSMD3 somatic mutations have been reported in HBV-associated HCC with portal vein tumor thrombosis and intrahepatic metastasis.^47^ To date, the physiological function of CSMD3 has not been extensively studied and its role in hepatocarcinogenesis has not been fully investigated. Further studies are needed to clarify its role in liver cancer.

When examining the regulatory cascade from pre-S/S-sW172* to *CSMD3* suppression, it was found that activation of miR-873 only occurred in Hepa1-6 and HepY2 cells, suggesting that other cellular factors were involved in this process. Interestingly, following miR-873 upregulation, GRP78 expression was enhanced in most of the hepatoma cell lines. As such, the increased ER stress in transgenic mice not only caused by pre-S/S-sW172* accumulation but also by miR-873 activation. Notably, in almost all cell lines (except for Huh7 and HepY2), miR-873 activation led to enhanced cell proliferation, consistent with the view that its target, *CSMD3*, was a tumor suppressor gene. At this time, the reason for the paradoxical growth regulation effect in HepY2 cells was unclear.

In summary, we provided transgenic mouse evidence showing an increased oncogenicity of truncated HBV pre-S/S protein with sW172* mutation. Besides increased ER stress response and apoptosis, resulting in enhanced hepatocyte proliferation and turnover, a miR-873-mediated downregulation of CSMD3 contributed partly to hepatocarcinogenesis.

## Materials and methods

### Construction of transgenic mice

The *Bgl*II to *Bgl*II HBV-DNA fragment (nt. 2434–1990) was isolated from pCMV-HBV^48, 49^ by restriction enzyme digestion (*Bgl*II (nt. 2434 in HBV genome) to *Xba*I (multiple cloning site of pRc/CMV, Invitrogen)) and was used to replace the *Bgl*II to *Xba*I region of pRc/CMV. This replacement also deleted the region containing CMV promoter. As a result, the final product, pTg-WT, encoded the whole pre-S/S gene driven by its native promoters but not CMV promoter. To construct the plasmid encoding the rtA181T/sW172* mutation, site-directed mutagenesis was performed to generate the desired point mutation, G673A, as described previously.^31^ Following the same procedure, the resulting plasmid, pTg-sW172*, was the same as pTg-WT except for the presence of target mutation (Figure 1a).

The transgenic mice were generated by pronucleus microinjection of C57BL/6 fertilized egg.^50^ All mice were maintained in a specific pathogen free facility in Chang Gung Medical Center, Linkou, Taiwan. The tails of mice were cut at 3 weeks of age and genomic DNA was isolated by use of the proteinase K digestion followed by phenol/chloroform extraction method as described previously.^31^ The success of gene transfer was determined by PCR using primers franking the target mutation: 5′-TGTGTCTGCGGCGTTTTATC-3′ (nt. 381–400; sense) and 5′-GTTTAAATGTATACCCAGAGAC-3′ (nt. 840–819; antisense). The resulting PCR product was sent for nucleotide sequencing to confirm the genotype. RT-PCR was also performed using these primers to calculate the copy number of transgene in each line.

### Maintenance of transgenic mice

Four lines of transgenic mice were maintained in a specific pathogen free facility to meet their growth conditions under the approval of Institutional Animal Care and Use Committee, Chang Gung Memorial Hospital, Taiwan. Regular genotyping, serum HBV surface antigen (HBsAg) testing, liver biochemistry, histology and final sacrifice at 18 months old for observation of tumor formation were performed. Quantitative assessment of HBsAg was performed using Elecsys HBsAg II Quant assay (Roche Diagnostics, Indianapolis, IN, USA).

The sample size used to calculate the incidence of HCC for each line of transgenic mice was selected to ensure that development of HCC in >15% of TgSW172* mice could reach statistical significance. All mice were examined by liver biopsy for tissue HBsAg expression by IHC method. Transgenic mice that did not express HBsAg were excluded for calculation. No randomization process was involved in this study. No blinding process was performed in this study.

### Western blot, immunohistochemistry analysis and TUNEL assay

For confirmation of HBV pre-S/S protein expression, monoclonal anti-HBs antibody (M-21853, Genzyme Diagnostics, San Carlos, CA, USA), goat polyclonal anti-HBs antibody (bs-1557G, Bioss, Woburn, MA, USA), monoclonal anti-pre-S2 antibody (sc-23944, Santa Cruz Biotechnology, Dallas, TX, USA) were used in western blot or IHC analysis. For characterization of HCC and oncogenic molecular pathway, anti-glypican-3 antibody (ab66596, Abcam, Cambridge, MA, USA), anti-GRP78 antibody (ab108615, Abcam, Cambridge), anti-ERK1/2 antibody (137F5, Cell Signaling Technology), anti-phosphorylated extracellular signal-regulated kinases 1/2 antibody (20G11, Cell Signaling Technology), β-actin antibody (GeneTex, Irvine, CA, USA), anti-B-cell lymphoma-extra large antibody (54H6, Cell Signaling Technology), anti-cyclin E antibody (sc-481, Santa Cruz Biotechnology), anti-CSMD3 antibody (sc-68281, Santa Cruz Biotechnology) and anti-GAPDH antibody (GeneTex) were used. Cell proliferation was also assessed using anti-Ki-67 antibody (Merck Millipore, Temecula, CA, USA). Apoptotic cells in liver tissue sections were detected using the DeadEnd Fluorometric TUNEL System (Promega Corporation, Madison, MI, USA) according to the manufacturer's manual. Cell apoptosis was also assessed using anti-activated caspase 3 antibody (EnoGene Biotech, New York, NY, USA).

### Cell lines

Hepa1-6, BNL, SK-Hep-1, Huh7, J7 and Mahlavu cells were maintained in Gibco Dulbecco's Modified Eagle Medium supplemented with 5% fetal bovine serum. HepG2 cells were maintained in Minimal Essential Medium supplemented with 5% fetal bovine serum. Hep-Y2 cells were maintained in RPMI medium 1640 with 5% fetal bovine serum. All culture medium was purchased from Life Technologies, Carlsbad, CA, USA. All cell lines were purchased from American Type Culture Collection except for Hep-Y2 cells, which were developed in our laboratory.^51^ All cell lines were tested negative for mycoplasma contamination.

### cDNA microarray

Three liver tissues from the noncancerous parts of negative control (Null; non-transgenic), TgWT-H, TgSW172*-H and TgSW172*-L mice were collected for cDNA microarray experiments. Total RNA was isolated from liver tissues using Trizol (Invitrogen Corporation, Carlsbad, CA, USA). The cDNA microarray system used in this study was GeneChip Gene 1.0 ST Array System for Mouse by Affymetrix which interrogated 28,853 well-annotated genes with 770,317 distinct probes.

### microRNA array

The microRNA array system used in this study was GeneChip miRNA 2.0 Array by Affymetrix which contained 100 percent miRBase v15 coverage, 15,644 probe sets, 2334 snoRNAs and scaRNAs and 2202 probe sets unique to pre-miRNA hairpin sequences. Total RNA was isolated from liver tissues using Trizol (Invitrogen Corporation). All microarray data were submitted to the GEO web site of NCBI (https://submit.ncbi.nlm.gov/geo/submission).

### Gene expression microarray data analysis

The fluorescent signals of the microarrays were scanned as digital images and then converted and summarized to intensity readings per probe set using Affymetrix expression console. The intensity readings were normalized using robust multiarray average algorithm. Gene expression levels were compared across groups using *t*-test, ANOVA, Mann–Whitney tests, fold-change comparison or regression-based methods. The genes with significant changes were visualized as heatmaps using Cluster 3.0 and TreeView. The annotation of the genes was analyzed using Affymetrix NetAffx, Metacore and/or NIH/DAVID.

### RT-qPCR validation of gene expression

The highest or critical up- and down-regulated genes from microarray experiments were validated by quantitative reverse transcription PCR (RT-qPCR). Briefly, the first-strand cDNA was synthesized from 0.5 μg of total RNA using SuperScript III First-Strand Synthesis System (Invitrogen, Carlsbad, CA), followed by PCR using the SYBR Green PCR Master Mix (Life Technologies Co., Carlsbad, CA). Primers used for RT-qPCR are listed in Table 2. For microRNA quantification, the NCode VILO miRNA cDNA Synthesis Kit and NCode EXPRESS SYBRR GreenER miRNA qRT PCR Kit (Life Technologies Co., Carlsbad, CA) were used according to the manufacturer's protocol.

### Inhibition of microRNA-873 expression

To inhibit miR-873 expression in human and murine hepatoma cell lines, miR-873 inhibition plasmid was purchased. The plasmid was generated based on a lentivirus expression vector, TOOLSilent shRNA Vector (TOOLS Biotechnology, New Taipei City, Taiwan), with insertion of a short stretch DNA sequence complementary to a target area of the miR-873 stem-loop sequence. The targeted sequence of miR-873 was 5'-AGGAGACTCACAAGTTCCTG C-3'. This vector also contained a green fluorescent protein gene under control of cytomegalovirus promoter for transfection efficiency monitoring.

### Measurement of miR-873 and anti-miR-873 levels

A stem-loop reverse transcription-quantitative real-time PCR (RT-qPCR) method was carried out as described previously.^52^ The qPCR reactions were conducted using ABI 7500 (Applied Biosystems, Foster City, CA), and the threshold cycle (Ct) and relative quantification was calculated by ABI SDS v1.4 software. The Ct was defined as the cycle number at which fluorescence was determined to be statistically significant above background. For measuring the miRNA expression level from cells transfected with antisense miR-873-5p or miR-873 mimics, the ΔCt value was used to calculate expression levels normalized against U6 RNA. The anti-miR-873-5p and miR-873-5p primer sequences used were as follows: anti-miR-873-5p RT primer, 5'-CTCAACTGGTGTCGTGGAGTCGGCAATTCAGTTGAGGCAGGAAC-3' anti-miR-873-5p specific (forward) primer, 5'-CGGCGGAGAGGAGACTCACGTT-3' miR-873-5p RT primer, 5'-CTCAACTGGTGTCGTGGAGTCGGCAATTCAGTTGAGAGGAGACT-3' miR-873-5p specific (forward) primer, 5'-CGGCGGGCAGGAACTTGTGAGT-3' universal reverse primer, 5'-CTGGTGTCGTGGAGTCGGCAATTC-3'.

### 3'-UTR luciferase reporter assay

Full-length *CSMD3* 3'UTRs of human (nt. 10 648–12 486; GenBank accession number AY210419) and mouse (nt. 11 210–13 022; GenBank accession number NM_001081391) were amplified by PCR respectively and cloned into a pMIR-REPORT vector (Applied Biosystems) carrying the luciferase reporter gene. The PCR primers used were: forward (human), 5'-TGAGCTCCGAGGCAACCTTTGCCTT-3' (with SacI site); reverse (human), 5'- GGCGACGCGTTTTTACAAAAGCAATTATCC-3' (with MluI site); forward (mouse), 5'-AATGAGCTCCACGGCAACACGTTGC-3' (with SacI site); and reverse (mouse), 5'-GCGG ACGCGTCAAAAGCAATTATCCTTTT-3' (with MluI site). The putative miR-873-5p recognition seed site in the *CSMD* 3'UTR were subjected to site-specific mutagenesis by QuikChange Multi Site-Directed Mutagenesis Kit (Agilent Technologies, Santa Clara, CA, USA) (*CSMD* 3'UTR mut), and the mutated sequences were validated via automated DNA sequencing. To examine the effects of miR-873 on *CSMD3* 3'UTR, the pMIR-CSMD3-3'UTR (wt or mut) was co-transfected with pre-miR-873 or mock control vector into hepatoma cells. Cells were harvested 72 h after transfection and cell extracts were submitted for luciferase activity assays according to the manufacture's protocol (Dual-Luciferase Reporter Assay System, Promega).

### Statistical analysis

Except for the cDNA microarray and microRNA array, all other statistical comparison was performed using student's *t*-test with >5 samples in each group. All the data met the criteria of normal distribution. Variance in each group was estimated to ensure there was no significant difference.

## Figures and Tables

**Table 1:** Incidence of hepatic tumors in HBV pre-S/S gene transgenic mice

*Strain*	*Characteristics*	*Sex*	*Number*	*Mice with liver tumor*	*Incidence*
TgWT-L	Serum HBsAg=419±162 IU/ml^a^				
		Male	16	0	0.0%
		Female	11	0	0.0%
		Combined	27	0	0.0%
TgWT-H	Serum HBsAg=599±159 IU/ml^a^				
		Male	17	0	0.0%
		Female	10	0	0.0%
		Combined	27	0	0.0%
TgSW172*-L	Lower levels of intrahepatic truncated surface proteins. Serum HBsAg undetectable				
		Male	15	1	6.7%
		Female	9	1	11.1%
		Combined	24	2	8.3%^b^
TgSW172*-H	Higher levels of intrahepatic truncated surface proteins. Serum HBsAg=10.2±19.1 IU/ml				
		Male	11	2	18.2%
		Female	15	4	26.7%
		Combined	26	6	23.1%^b,c^

a The two concentrations were significantly different, *P*=0.0001.

b *P*=0.0021, when the two mutant type TgSW172*-L and TgSW172*-H mice were combined and compared with the two wild type TgWT-L and TgWT-H mice combined.

c *P*=0.0008, when the TgSW172*-H mice were compared with the two wild type TgWT-L and TgWT-H mice combined.

**Table 2:** Primers designed for the RT-qPCR assay in the validation study.

*Gene*	*Forward primer*	*Reverse primer*
Mecp2	CTTCTGTAGACCAGCTCCAAC	GTTGTAGTGGCTCATGCTTGC
Appl1	CCAGCCTTCGCCACGAT	TCGAAGTCAGGTGTGTTGCT
ZFHX4	GCGCACTCAACTTAATCAGCC	TCCTACTCGATCTTCGCCTC
BCL2L2	GGCGGAGTTCACAGCTCTAT	CTGTTCCGTGACCATCCAGT
IRF2BP2	GCCAGTCGTGCTATCTGTGT	GTAGCGCTCCATAGCCTGAG
PID1	TCTCTACCACAGGCATGCAG	ATGGCCGGATCTCTAGGAGG
PPARGC1A	CACTACAGACACCGCACACA	ATCACACGGCGCTCTTCAAT
CSMD3	AACAGCAACAACTCTACTGCG	AGGATGAAGTCTAGGCGGC
ATP8B2	CATGGATTGCTGGAACCTTGCT	AAGGCACAATGGTGGTGAAC
TANC2	GTGCGGATCCTCCGTGG	GGTTCCTCGCTTCCTCCATC
